# 3-(4-Fluoro­phenyl­sulfon­yl)-2-methyl­naphtho­[1,2-*b*]furan

**DOI:** 10.1107/S1600536810048361

**Published:** 2010-11-27

**Authors:** Hong Dae Choi, Pil Ja Seo, Byeng Wha Son, Uk Lee

**Affiliations:** a Department of Chemistry, Dongeui University, San 24 Kaya-dong Busanjin-gu, Busan 614-714, Republic of Korea; b Department of Chemistry, Pukyong National University, 599-1 Daeyeon 3-dong, Nam-gu, Busan 608-737, Republic of Korea

## Abstract

In the title compound, C_19_H_13_FO_3_S, the 4-fluoro­phenyl ring makes a dihedral angle of 68.59 (5)° with the mean plane of the naphtho­furan fragment. In the crystal, mol­ecules are linked by weak inter­molecular C—H⋯O, C—H⋯F and C—H⋯π inter­actions. The crystal structure also exhibits aromatic π–π inter­actions between the central benzene and the outer benzene rings of neighbouring mol­ecules [centroid–centroid distance = 3.650 (3) Å].

## Related literature

For the pharmacological activity of naphtho­furan compounds, see: Einhorn *et al.* (1984 ▶); Hranjec *et al.* (2003 ▶); Mahadevan & Vaidya (2003 ▶). For our previous structural studies of related 3-aryl­sulfonyl-2-methyl­naphtho­[1,2-*b*]furan derivatives, see: Choi *et al.* (2008**a* ▶,b* ▶).
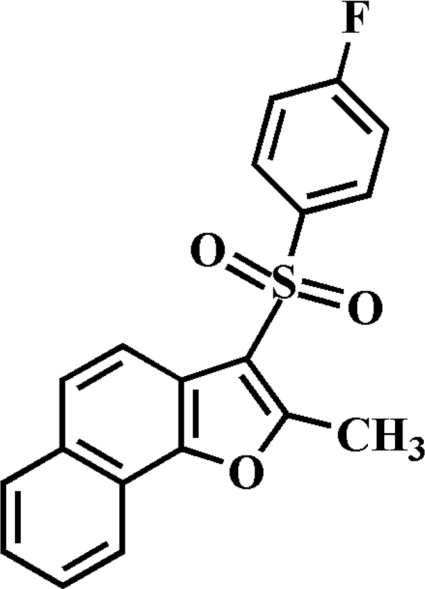


## Experimental

### 

#### Crystal data

C_19_H_13_FO_3_S
*M* *_r_* = 340.35
Orthorhombic, 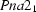

*a* = 8.1456 (3) Å
*b* = 18.4472 (5) Å
*c* = 10.3618 (4) Å
*V* = 1557.00 (9) Å^3^
*Z* = 4
Mo *K*α radiation
μ = 0.23 mm^−1^
*T* = 173 K
0.30 × 0.25 × 0.12 mm

#### Data collection

Bruker SMART APEXII CCD diffractometer
Absorption correction: multi-scan (*SADABS*; Bruker, 2009 ▶) *T*_min_ = 0.664, *T*_max_ = 0.746
7603 measured reflections
2719 independent reflections
2502 reflections with *I* > 2σ(*I*)
*R*_int_ = 0.026

#### Refinement

*R*[*F* ^2^ > 2σ(*F* ^2^)] = 0.030
*wR*(*F* ^2^) = 0.075
*S* = 1.05
2719 reflections
218 parameters
1 restraint
H-atom parameters constrained
Δρ_max_ = 0.25 e Å^−3^
Δρ_min_ = −0.23 e Å^−3^
Absolute structure: Flack (1983 ▶), 830 Friedel pairs
Flack parameter: 0.09 (7)

### 

Data collection: *APEX2* (Bruker, 2009 ▶); cell refinement: *SAINT* (Bruker, 2009 ▶); data reduction: *SAINT*; program(s) used to solve structure: *SHELXS97* (Sheldrick, 2008 ▶); program(s) used to refine structure: *SHELXL97* (Sheldrick, 2008 ▶); molecular graphics: *ORTEP-3* (Farrugia, 1997 ▶) and *DIAMOND* (Brandenburg, 1998 ▶); software used to prepare material for publication: *SHELXL97*.

## Supplementary Material

Crystal structure: contains datablocks global, I. DOI: 10.1107/S1600536810048361/sj5062sup1.cif

Structure factors: contains datablocks I. DOI: 10.1107/S1600536810048361/sj5062Isup2.hkl

Additional supplementary materials:  crystallographic information; 3D view; checkCIF report

## Figures and Tables

**Table 1:** Hydrogen-bond geometry (Å, °) *Cg*1 is the centroid of the C14–C19 4-fluoro­phenyl ring.

*D*—H⋯*A*	*D*—H	H⋯*A*	*D*⋯*A*	*D*—H⋯*A*
C8—H8⋯O3^i^	0.95	2.46	3.379 (2)	163
C15—H15⋯F1^ii^	0.95	2.53	3.150 (3)	123
C16—H16⋯O3^iii^	0.95	2.44	3.380 (2)	170
C4—H4⋯*Cg*1^iv^	0.95	2.75	3.625 (3)	154

Symmetry codes: (i) 
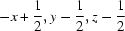
; (ii) 
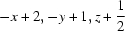
; (iii) 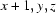; (iv) 
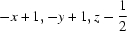
.

**Fig. 1.**
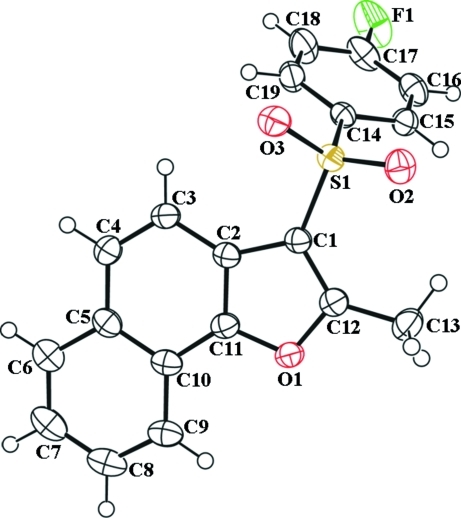
The molecular structure of the title compound with the atom numbering scheme. Displacement ellipsoids are drawn at the 50% probability level. H atoms are presented as a small spheres of arbitrary radius.

**Fig. 2.**
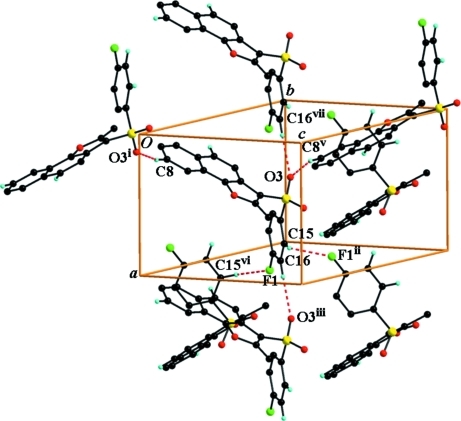
A view of C–H···O and C–H···F interactions (dotted lines) in the crystal structure of the title compound. [Symmetry codes: (i) - x +1/2, y - 1/2, z - 1/2; (ii) - x + 2, - y + 1, z + 1/2; (iii) x + 1, y, z; (v) - x +1/2, y + 1/2, z + 1/2; (vi) - x + 2, - y + 1, z - 1/2 ; (vii) x - 1, y, z.]

**Fig. 3.**
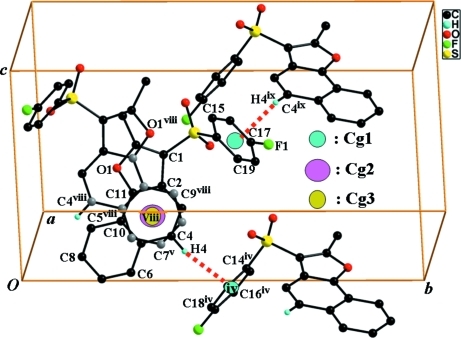
A view of C–H···π and π–π interactions (dotted lines) in the crystal structure of the title compound. [Symmetry codes: (iv) - x + 1, - y + 1, z - 1/2 ; (viii) x + 1/2, - y + 1/2, z; (ix) - x + 1, - y + 1 , z + 1/2.]

## supplementary crystallographic information

### Comment

Many compounds containing a naphthofuran moiety show diverse pharmacological properties such as antibacterial, antitumor and anthelmintic activities (Einhorn *et al.*, 1984, Hranjec *et al.*, 2003, Mahadevan & Vaidya, 2003). As a part of our ongoing studies of the substituent effect on the solid state structures of 3-arylsulfonyl-2-methylnaphtho[1,2-b]furan analogues (Choi *et al.*, 2008**a*,b*), we report herein on the crystal structure of the title compound.

In the title molecule (Fig. 1), the naphthofuran unit is essentially planar, with a mean deviation of 0.008 (2) Å from the least-squares plane defined by the thirteen constituent atoms. The dihedral angle formed by the mean plane of the naphthofuran ring and the 4-fluorophenyl ring is 68.59 (5)°. The crystal packing (Fig. 2) is stabilized by weak intermolecular C–H···O and C–H···F hydrogen bonds; the first one between a benzene H atom and the oxygen of the O═S═O unit (Table 1; C8–H8···O3^i^), and the second one between the 4–fluorophenyl H atom and the oxygen of the O═S═O unit (Table 1; C16–H16···O3^iii^), and the third one between the 4-fluorophenyl H atom and the fluorine (Table 1; C15–H15···F1^ii^). The crystal packing (Fig. 3) is also exhibits an intermolecular C–H..π interaction between a benzene H atom and the 4-fluorophenyl ring (Table 1; C4–H4···Cg1^iv^, Cg1 is the centroid of the C14-C19 4-fluorophenyl ring). The crystal packing (Fig. 3) is further stabilized by an aromatic π–π interaction between the central benzene and the outer benzene rings of neighbouring molecules. The Cg2···Cg3^viii^ distance is 3.650 (3) Å (Cg2 and Cg3 are the centroids of the C2/C3/C4/C5/C10/C11 benzene ring and the C5-C10 benzene ring, respectively).

### Experimental

77% 3-chloroperoxybenzoic acid (493 mg, 2.2 mmol) was added in small portions to a stirred solution of 3-(4-fluorophenylsulfanyl)-2-methylnaphtho [1,2-b]furan (339 mg, 1.1 mmol) in dichloromethane (30 mL) at 273 K. After being stirred at room temperature for 8h, the mixture was washed with saturated sodium bicarbonate solution and the organic layer was separated, dried over magnesium sulfate, filtered and concentrated at reduced pressure. The residue was purified by column chromatography (hexane-ethyl acetate, 4:1 v/v) to afford the title compound as a colorless solid [yield 71%, m.p. 439-440 K; *R*_f_ = 0.55 (hexane-ethyl acetate, 4:1 v/v)]. Single crystals suitable for X-ray diffraction were obtained by slow evaporation of a solution of the title compound in acetone at room temperature.

### Refinement

All H atoms were positioned geometrically and refined using a riding model, with C–H = 0.95 Å for aryl and 0.98 Å for methyl H atoms. *U*_iso_(H) = 1.2*U*_eq_(C) for aryl and 1.5*U*_eq_(C) for methyl H atoms.

### Crystal data

**Table:**

C_19_H_13_FO_3_S	*F*(000) = 704
*M**_r_* = 340.35	*D*_x_ = 1.452 Mg m^−^^3^
Orthorhombic, *P**n**a*2_1_	Mo *K*α radiation, λ = 0.71073 Å
Hall symbol: P 2c -2n	Cell parameters from 3161 reflections
*a* = 8.1456 (3) Å	θ = 2.3–27.5°
*b* = 18.4472 (5) Å	µ = 0.23 mm^−^^1^
*c* = 10.3618 (4) Å	*T* = 173 K
*V* = 1557.00 (9) Å^3^	Block, colourless
*Z* = 4	0.30 × 0.25 × 0.12 mm

### Data collection

**Table:**

Bruker SMART APEXII CCD diffractometer	2719 independent reflections
Radiation source: rotating anode	2502 reflections with *I* > 2σ(*I*)
graphite multilayer	*R*_int_ = 0.026
Detector resolution: 10.0 pixels mm^-1^	θ_max_ = 27.5°, θ_min_ = 2.2°
φ and ω scans	*h* = −10→4
Absorption correction: multi-scan (*SADABS*; Bruker, 2009)	*k* = −23→22
*T*_min_ = 0.664, *T*_max_ = 0.746	*l* = −10→13
7603 measured reflections	

### Refinement

**Table:**

Refinement on *F*^2^	Secondary atom site location: difference Fourier map
Least-squares matrix: full	Hydrogen site location: difference Fourier map
*R*[*F*^2^ > 2σ(*F*^2^)] = 0.030	H-atom parameters constrained
*wR*(*F*^2^) = 0.075	*w* = 1/[σ^2^(*F*_o_^2^) + (0.0405*P*)^2^ + 0.1905*P*] where *P* = (*F*_o_^2^ + 2*F*_c_^2^)/3
*S* = 1.05	(Δ/σ)_max_ = 0.001
2719 reflections	Δρ_max_ = 0.25 e Å^−^^3^
218 parameters	Δρ_min_ = −0.23 e Å^−^^3^
1 restraint	Absolute structure: Flack (1983), 830 Friedel pairs
Primary atom site location: structure-invariant direct methods	Flack parameter: 0.09 (7)

### Special details

**Table:**

Geometry. All esds (except the esd in the dihedral angle between two l.s. planes) are estimated using the full covariance matrix. The cell esds are taken into account individually in the estimation of esds in distances, angles and torsion angles; correlations between esds in cell parameters are only used when they are defined by crystal symmetry. An approximate (isotropic) treatment of cell esds is used for estimating esds involving l.s. planes.
Refinement. Refinement of F^2^ against ALL reflections. The weighted R-factor wR and goodness of fit S are based on F^2^, conventional R-factors R are based on F, with F set to zero for negative F^2^. The threshold expression of F^2^ > 2sigma(F^2^) is used only for calculating R-factors(gt) etc. and is not relevant to the choice of reflections for refinement. R-factors based on F^2^ are statistically about twice as large as those based on F, and R- factors based on ALL data will be even larger.

### Fractional atomic coordinates and isotropic or equivalent isotropic displacement parameters (Å^2^)

**Table:**

	*x*	*y*	*z*	*U*_iso_*/*U*_eq_	
S1	0.52554 (5)	0.41581 (2)	0.52995 (6)	0.02619 (12)	
F1	1.07897 (19)	0.56345 (10)	0.29185 (19)	0.0694 (5)	
O1	0.46264 (16)	0.22234 (6)	0.38327 (16)	0.0295 (3)	
O2	0.57654 (19)	0.39444 (8)	0.65655 (16)	0.0367 (4)	
O3	0.38342 (15)	0.46132 (7)	0.51578 (19)	0.0351 (4)	
C1	0.4869 (2)	0.33848 (9)	0.4394 (2)	0.0256 (4)	
C2	0.3939 (2)	0.33602 (9)	0.3211 (2)	0.0262 (4)	
C3	0.3171 (2)	0.38737 (10)	0.2398 (2)	0.0294 (5)	
H3	0.3221	0.4377	0.2585	0.035*	
C4	0.2358 (2)	0.36252 (10)	0.1336 (2)	0.0321 (5)	
H4	0.1835	0.3965	0.0784	0.039*	
C5	0.2264 (2)	0.28691 (11)	0.1021 (2)	0.0316 (5)	
C6	0.1433 (3)	0.26263 (12)	−0.0094 (2)	0.0399 (6)	
H6	0.0923	0.2967	−0.0653	0.048*	
C7	0.1359 (3)	0.18989 (13)	−0.0374 (3)	0.0475 (6)	
H7	0.0807	0.1740	−0.1130	0.057*	
C8	0.2087 (3)	0.13917 (11)	0.0444 (3)	0.0447 (6)	
H8	0.2016	0.0891	0.0240	0.054*	
C9	0.2901 (3)	0.16034 (10)	0.1534 (3)	0.0371 (5)	
H9	0.3390	0.1253	0.2084	0.044*	
C10	0.3008 (2)	0.23490 (9)	0.1835 (2)	0.0294 (5)	
C11	0.3834 (2)	0.26340 (9)	0.2913 (2)	0.0276 (4)	
C12	0.5250 (2)	0.26895 (10)	0.4729 (2)	0.0286 (4)	
C13	0.6087 (3)	0.23448 (11)	0.5836 (3)	0.0382 (5)	
H13A	0.6786	0.1949	0.5524	0.057*	
H13B	0.5266	0.2151	0.6435	0.057*	
H13C	0.6764	0.2706	0.6281	0.057*	
C14	0.6915 (2)	0.45978 (9)	0.4545 (2)	0.0251 (4)	
C15	0.8504 (2)	0.44194 (10)	0.4931 (2)	0.0304 (5)	
H15	0.8679	0.4059	0.5572	0.036*	
C16	0.9823 (3)	0.47724 (12)	0.4372 (3)	0.0386 (5)	
H16	1.0917	0.4664	0.4624	0.046*	
C17	0.9511 (3)	0.52791 (13)	0.3451 (3)	0.0423 (6)	
C18	0.7955 (3)	0.54585 (13)	0.3041 (3)	0.0455 (6)	
H18	0.7794	0.5813	0.2388	0.055*	
C19	0.6633 (3)	0.51109 (11)	0.3605 (2)	0.0359 (5)	
H19	0.5544	0.5224	0.3346	0.043*	

### Atomic displacement parameters (Å^2^)

**Table:**

	*U*^11^	*U*^22^	*U*^33^	*U*^12^	*U*^13^	*U*^23^
S1	0.0226 (2)	0.02804 (19)	0.0279 (3)	0.00002 (15)	0.0020 (3)	−0.0023 (2)
F1	0.0527 (9)	0.1072 (12)	0.0483 (10)	−0.0428 (9)	0.0082 (9)	0.0102 (10)
O1	0.0281 (7)	0.0237 (6)	0.0368 (9)	0.0001 (5)	0.0003 (6)	0.0019 (6)
O2	0.0381 (8)	0.0429 (7)	0.0291 (9)	−0.0014 (6)	0.0014 (8)	0.0032 (7)
O3	0.0239 (6)	0.0338 (6)	0.0477 (11)	0.0044 (5)	0.0033 (8)	−0.0078 (8)
C1	0.0223 (8)	0.0251 (8)	0.0293 (12)	−0.0009 (6)	0.0016 (9)	−0.0008 (9)
C2	0.0229 (9)	0.0256 (8)	0.0301 (12)	−0.0012 (7)	0.0028 (9)	−0.0025 (8)
C3	0.0295 (10)	0.0241 (8)	0.0344 (13)	−0.0008 (7)	0.0016 (9)	−0.0006 (8)
C4	0.0329 (10)	0.0325 (8)	0.0310 (13)	0.0016 (8)	0.0001 (10)	0.0035 (9)
C5	0.0257 (10)	0.0358 (10)	0.0332 (12)	−0.0049 (8)	0.0040 (9)	−0.0025 (9)
C6	0.0381 (11)	0.0440 (12)	0.0375 (14)	−0.0043 (9)	0.0003 (10)	−0.0036 (10)
C7	0.0465 (13)	0.0545 (14)	0.0416 (15)	−0.0139 (10)	0.0016 (13)	−0.0153 (12)
C8	0.0481 (12)	0.0354 (9)	0.0506 (17)	−0.0112 (9)	0.0099 (14)	−0.0125 (12)
C9	0.0361 (11)	0.0291 (9)	0.0459 (15)	−0.0062 (8)	0.0074 (11)	−0.0035 (10)
C10	0.0263 (9)	0.0275 (9)	0.0346 (12)	−0.0032 (7)	0.0077 (9)	−0.0040 (8)
C11	0.0239 (9)	0.0252 (8)	0.0338 (11)	−0.0008 (7)	0.0039 (9)	0.0013 (8)
C12	0.0227 (9)	0.0297 (9)	0.0335 (12)	−0.0007 (7)	0.0036 (9)	0.0019 (9)
C13	0.0352 (11)	0.0347 (10)	0.0448 (15)	0.0048 (9)	−0.0024 (11)	0.0075 (10)
C14	0.0219 (8)	0.0271 (8)	0.0263 (11)	−0.0021 (7)	0.0002 (8)	−0.0029 (8)
C15	0.0263 (9)	0.0306 (9)	0.0343 (13)	0.0027 (7)	−0.0029 (8)	−0.0025 (8)
C16	0.0252 (9)	0.0505 (12)	0.0400 (15)	−0.0028 (8)	−0.0008 (10)	−0.0093 (12)
C17	0.0389 (12)	0.0574 (13)	0.0307 (14)	−0.0206 (10)	0.0064 (11)	−0.0057 (12)
C18	0.0508 (14)	0.0525 (12)	0.0333 (14)	−0.0185 (11)	−0.0082 (12)	0.0124 (12)
C19	0.0355 (11)	0.0384 (9)	0.0339 (13)	−0.0074 (8)	−0.0086 (10)	0.0057 (10)

### Geometric parameters (Å, °)

**Table:**

S1—O2	1.4313 (18)	C7—H7	0.9500
S1—O3	1.4375 (13)	C8—C9	1.366 (4)
S1—C1	1.736 (2)	C8—H8	0.9500
S1—C14	1.7596 (19)	C9—C10	1.413 (2)
F1—C17	1.348 (2)	C9—H9	0.9500
O1—C12	1.364 (3)	C10—C11	1.406 (3)
O1—C11	1.378 (3)	C12—C13	1.478 (3)
C1—C12	1.365 (3)	C13—H13A	0.9800
C1—C2	1.442 (3)	C13—H13B	0.9800
C2—C11	1.377 (2)	C13—H13C	0.9800
C2—C3	1.414 (3)	C14—C19	1.378 (3)
C3—C4	1.364 (3)	C14—C15	1.394 (3)
C3—H3	0.9500	C15—C16	1.384 (3)
C4—C5	1.434 (3)	C15—H15	0.9500
C4—H4	0.9500	C16—C17	1.360 (4)
C5—C6	1.412 (3)	C16—H16	0.9500
C5—C10	1.414 (3)	C17—C18	1.377 (3)
C6—C7	1.374 (3)	C18—C19	1.382 (3)
C6—H6	0.9500	C18—H18	0.9500
C7—C8	1.395 (4)	C19—H19	0.9500
			
O2—S1—O3	119.22 (11)	C11—C10—C9	124.7 (2)
O2—S1—C1	108.75 (9)	C11—C10—C5	115.16 (16)
O3—S1—C1	106.18 (9)	C9—C10—C5	120.2 (2)
O2—S1—C14	108.11 (10)	C2—C11—O1	110.52 (19)
O3—S1—C14	107.71 (9)	C2—C11—C10	124.85 (19)
C1—S1—C14	106.15 (10)	O1—C11—C10	124.62 (15)
C12—O1—C11	107.39 (14)	O1—C12—C1	109.55 (19)
C12—C1—C2	107.83 (17)	O1—C12—C13	115.41 (16)
C12—C1—S1	126.42 (18)	C1—C12—C13	135.0 (2)
C2—C1—S1	125.49 (14)	C12—C13—H13A	109.5
C11—C2—C3	119.37 (19)	C12—C13—H13B	109.5
C11—C2—C1	104.71 (18)	H13A—C13—H13B	109.5
C3—C2—C1	135.91 (17)	C12—C13—H13C	109.5
C4—C3—C2	118.08 (16)	H13A—C13—H13C	109.5
C4—C3—H3	121.0	H13B—C13—H13C	109.5
C2—C3—H3	121.0	C19—C14—C15	121.31 (19)
C3—C4—C5	122.4 (2)	C19—C14—S1	120.18 (15)
C3—C4—H4	118.8	C15—C14—S1	118.50 (16)
C5—C4—H4	118.8	C16—C15—C14	119.3 (2)
C6—C5—C10	118.56 (18)	C16—C15—H15	120.3
C6—C5—C4	121.3 (2)	C14—C15—H15	120.3
C10—C5—C4	120.1 (2)	C17—C16—C15	118.2 (2)
C7—C6—C5	120.2 (2)	C17—C16—H16	120.9
C7—C6—H6	119.9	C15—C16—H16	120.9
C5—C6—H6	119.9	F1—C17—C16	118.5 (2)
C6—C7—C8	120.6 (2)	F1—C17—C18	117.9 (2)
C6—C7—H7	119.7	C16—C17—C18	123.6 (2)
C8—C7—H7	119.7	C17—C18—C19	118.4 (2)
C9—C8—C7	121.1 (2)	C17—C18—H18	120.8
C9—C8—H8	119.4	C19—C18—H18	120.8
C7—C8—H8	119.4	C14—C19—C18	119.2 (2)
C8—C9—C10	119.4 (2)	C14—C19—H19	120.4
C8—C9—H9	120.3	C18—C19—H19	120.4
C10—C9—H9	120.3		
			
O2—S1—C1—C12	−10.6 (2)	C1—C2—C11—C10	179.16 (19)
O3—S1—C1—C12	−140.05 (17)	C12—O1—C11—C2	−0.1 (2)
C14—S1—C1—C12	105.50 (18)	C12—O1—C11—C10	−179.04 (19)
O2—S1—C1—C2	162.76 (16)	C9—C10—C11—C2	−179.9 (2)
O3—S1—C1—C2	33.3 (2)	C5—C10—C11—C2	0.6 (3)
C14—S1—C1—C2	−81.12 (18)	C9—C10—C11—O1	−1.1 (3)
C12—C1—C2—C11	−0.3 (2)	C5—C10—C11—O1	179.34 (18)
S1—C1—C2—C11	−174.67 (15)	C11—O1—C12—C1	−0.1 (2)
C12—C1—C2—C3	178.4 (2)	C11—O1—C12—C13	177.31 (18)
S1—C1—C2—C3	4.0 (3)	C2—C1—C12—O1	0.2 (2)
C11—C2—C3—C4	−0.4 (3)	S1—C1—C12—O1	174.55 (14)
C1—C2—C3—C4	−178.9 (2)	C2—C1—C12—C13	−176.4 (2)
C2—C3—C4—C5	−0.2 (3)	S1—C1—C12—C13	−2.1 (3)
C3—C4—C5—C6	−179.1 (2)	O2—S1—C14—C19	−152.63 (17)
C3—C4—C5—C10	1.0 (3)	O3—S1—C14—C19	−22.6 (2)
C10—C5—C6—C7	−0.1 (3)	C1—S1—C14—C19	90.83 (18)
C4—C5—C6—C7	−180.0 (2)	O2—S1—C14—C15	26.78 (19)
C5—C6—C7—C8	0.6 (4)	O3—S1—C14—C15	156.85 (16)
C6—C7—C8—C9	−0.5 (4)	C1—S1—C14—C15	−89.76 (18)
C7—C8—C9—C10	−0.1 (3)	C19—C14—C15—C16	1.0 (3)
C8—C9—C10—C11	−178.9 (2)	S1—C14—C15—C16	−178.42 (17)
C8—C9—C10—C5	0.7 (3)	C14—C15—C16—C17	−0.6 (3)
C6—C5—C10—C11	179.01 (19)	C15—C16—C17—F1	178.8 (2)
C4—C5—C10—C11	−1.1 (3)	C15—C16—C17—C18	−0.2 (4)
C6—C5—C10—C9	−0.6 (3)	F1—C17—C18—C19	−178.3 (2)
C4—C5—C10—C9	179.30 (19)	C16—C17—C18—C19	0.7 (4)
C3—C2—C11—O1	−178.73 (17)	C15—C14—C19—C18	−0.5 (3)
C1—C2—C11—O1	0.2 (2)	S1—C14—C19—C18	178.88 (18)
C3—C2—C11—C10	0.2 (3)	C17—C18—C19—C14	−0.3 (4)

### Hydrogen-bond geometry (Å, °)

**Table:**

Cg1 is the centroid of the C14–C19 4-fluorophenyl ring.

**Table:**

*D*—H···*A*	*D*—H	H···*A*	*D*···*A*	*D*—H···*A*
C8—H8···O3^i^	0.95	2.46	3.379 (2)	163
C15—H15···F1^ii^	0.95	2.53	3.150 (3)	123
C16—H16···O3^iii^	0.95	2.44	3.380 (2)	170
C4—H4···Cg1^iv^	0.95	2.75	3.625 (3)	154

Symmetry codes: (i) −*x*+1/2, *y*−1/2, *z*−1/2; (ii) −*x*+2, −*y*+1, *z*+1/2; (iii) *x*+1, *y*, *z*; (iv) −*x*+1, −*y*+1, *z*−1/2.
